# Drug-Coated versus Uncoated Balloon for Side Branch Protection in Coronary Bifurcation Lesions Treated with Provisional Stenting Using Drug-Eluting Stents: A Meta-analysis

**DOI:** 10.1155/2022/5892589

**Published:** 2022-12-21

**Authors:** Zhi-Ming Jiang, Le Liu

**Affiliations:** ^1^Department of Cardiology, The Fourth Hospital of Changsha, Changsha, China; ^2^Department of Cardiology, The Eighth Hospital of Changsha, Changsha, China

## Abstract

**Background:**

Provisional stenting using drug-eluting stents (DES) has become the preferred treatment for coronary bifurcation lesions (CBLs). We performed a meta-analysis to compare the effects of side branch (SB) protection using a drug-coated balloon (DCB) versus an uncoated balloon (UCB) during the procedure.

**Methods:**

Relevant randomized and nonrandomized studies were identified by searching the Medline, Embase, Web of Science, Wanfang, and CNKI databases. We used a random-effect model to pool the data by incorporating the heterogeneity between the included studies.

**Results:**

Overall, 803 patients with CBLs treated with provisional stenting using DES were included from seven studies. With a follow-up duration of 6 to 12 months, SB protection with DCB was associated with a lower degree of postoperative diameter stenosis (mean difference (MD): −11.35%, 95% confidence interval (CI): −14.17 to−8.53, *p* < 0.001; I^2^ = 0%) and less late lumen loss (MD: −0.19 mm, 95% CI:−0.28 to−0.10, *p* < 0.001; I^2^ = 69%) of SB compared to those with UCB. Moreover, SB protection with DCB was associated with reduced risks of target lesion revascularization (risk ratio [RR]: 0.49, 95% CI: 0.27 to 0.88, *p* = 0.02; I^2^ = 0%) and major adverse cardiovascular events (RR: 0.42, 95% CI: 0.27 to 0.66, *p* < 0.01; I^2^ = 0%). Subgroup analysis according to the study design showed similar results.

**Conclusions:**

For patients with CBL treated with provisional stenting using DES, SB protection with DCB was associated with better angiographic and clinical outcomes than those with UCB.

## 1. Introduction

Coronary bifurcation lesions (CBLs) are coronary lesions commonly encountered by interventional cardiologists and account for up to 20% of all lesions treated with percutaneous coronary intervention (PCI) [1, 2]. More importantly, treatment for CBLs remains challenging, and the prognosis of patients after PCI needs to be improved [2, 3]. Currently, a provisional stenting strategy with drug-eluting stents (DES) is recommended as the standard procedure for the treatment of most CBLs [4, 5]. For this technique, the main vessel is implanted with DES, followed by kissing-balloon angioplasty and provisional stenting of the side branch (SB). Accumulating evidence suggests that a provisional stenting strategy was associated with better angiographic and clinical outcomes for the majority of CBLs as compared with the routine *T* stenting strategy [6–8]. For CBLs treated with provisional stenting strategy using DES, the proximal optimization technique (POT) is mandatory according to the recent consensus document of the European Bifurcation Club [9, 10]. This is important because the overall clinical prognosis of patients is not only determined by the status of the main vessel, but also by the lesions and treatments used for the SB.

Recent reports suggest that the use of a drug-coated balloon (DCB) to protect the SB during provisional stenting for CBLs is an attractive approach [11, 12]. DCB is a relatively new device category for PCI that involves the use of drug coatings to apply an anti-intimal hyperplasia effect to the surface of the balloon [13, 14]. During PCI, DCB releases the antiproliferative drugs onto the vessel wall while dilating the stenosis of the diseased vessel [15, 16]. The drug coating is rapidly absorbed by vascular wall tissue, facilitating the inhibition of intimal hyperplasia. The most common drug coating for DCB is paclitaxel, which can prevent restenosis and reduce cell differentiation by blocking the formation of microtubules [15, 16]. In addition, after balloon dilatation, the injury to the arterial wall leads to an inflammatory response, growth factor release, and smooth muscle cell migration [15, 16]. Paclitaxel can reduce the release of platelet-derived growth factors and inhibit the migration of vascular smooth muscle cells to the intima [15, 16]. Previous studies have confirmed the benefits of DCB for the treatment of in-stent restenosis [17] and small coronary artery lesion [18]. Besides, early case series have consistently shown that SB protection with DCB is safe and associated with favorable angiographic and short-term clinical outcomes [19, 20]. Subsequently, some clinical studies were performed to compare the effects of DCBs and conventional uncoated balloon (UCB) for CBLs treated with provisional stenting using DES [21–27]. Although most of the studies showed that DCB is superior to UCB in improving the angiographic outcomes for the SB, the benefits of DCB with regard to the clinical outcomes were rarely reported [21–27], probably due to the limited sample sizes of the studies. Therefore, in this study, we performed a meta-analysis of clinical studies to systematically evaluate the influence of SB protection with DCB compared with UCB on angiographic and clinical outcomes in patients with CBLs treated with provisional stenting using DES.

## 2. Methods

We followed the preferred reporting items for systematic reviews and meta-analyses (PRISMA) statement [28] and Cochrane Handbook [29] for the design, performance, and presentation of the meta-analysis.

### 2.1. Search of Electronic Databases

We identified studies through a systematic search of the Medline, Embase, Web of Science, China National Knowledge Infrastructure (CNKI), and Wanfang electronic databases using the following terms: (1) “drug-eluting balloon” OR “DEB” OR “drug-coated balloon” OR “DCB” OR “paclitaxel-coated balloon” OR “PCB” and (2) “bifurcation” OR “bifurcations”. Only clinical studies published in English or Chinese were selected. An additional manual check of the reference lists of relevant original and review articles was performed as a supplement. The last literature search was conducted on November 11, 2021.

### 2.2. Selection of Eligible Studies

The inclusion criteria were (1) randomized or nonrandomized controlled trials (RCTs or NRCTs) published as full-length articles, (2) included patients with CBLs treated with provisional stenting using DES, (3) patients were allocated to an interventional group of SB protection using DCB or a control group of SB protection using UCB, and (4) reported at least one of the following outcomes during follow-up: the extent of postoperative SB diameter stenosis, late lumen loss (LLL) of SB, incidence of target lesion revascularization (TLR) of SB, and incidence of major adverse cardiovascular events (MACEs). The difference between the minimum lumen diameters (MLDs) measured immediately after the procedure and at the angiographic follow-up was defined as LLL. The incidence of MACE was defined as a composite outcome of TLR, myocardial infarction, and cardiac death. Reviews, preclinical studies, studies that did not include patients with CBL, studies that did not apply provisional stenting using DES, or studies that did not report the outcomes of interest were excluded.

### 2.3. Extraction of Data and Evaluation of Study Quality

Two of the authors independently conducted electronic database searches, extraction of the study data, and assessment of the study quality according to the inclusion criteria described above. Discrepancies were resolved by consensus between the authors. The extracted data consisted of the following: (1) name of the first author, year of publication, study design, and country; (2) population characteristics, including total number of the patients, mean age, and sex; (3) procedure characteristics, including types of DES, DCB, and UCB were used; and (4) follow-up durations and outcomes reported. Quality evaluation of the RCTs was performed using Cochrane risk of bias tool [29] according to the following factors: (1) random sequence generation, (2) allocation concealment, (3) blinding of participants and personnel, (4) blinding of outcome assessors, (5) incomplete outcome data, (6) selective outcome reporting, and (7) other potential biases. The Newcastle–Ottawa scale (NOS) [30] was used for the quality assessment of NRCTs based on three domains: defining study groups, between-group comparability, and validation of the outcome. This scale was scored from 1 to 9 stars, with 9 stars indicating the highest study quality level.

### 2.4. Statistical Methods

Continuous variables were analyzed using the mean difference (MD), whereas dichotomous data were analyzed using risk ratios (RR), both with 95% confidence interval (CI). Cochrane's *Q* test was used to evaluate the heterogeneity, and the I^2^ statistic was also estimated [29]. Heterogeneity was deemed to be significant if I^2^ > 50% [31]. We used a random-effect model for data synthesis because the model incorporated the potential between-study heterogeneity and could provide a more generalized result [29]. Subgroup analyses were performed to determine whether the results were consistent for meta-analyses of RCTs and NRCTs. Funnel plots were constructed and a visual inspection of the symmetry was conducted to reflect the publication bias [32]. Egger's regression asymmetry test was further performed for the evaluation of potential publication bias [29]. We used the RevMan (Version 5.1; Cochrane Collaboration, Oxford, UK) software for the statistical analyses.

## 3. Results

### 3.1. Results of Database Search

**Figure 1:**
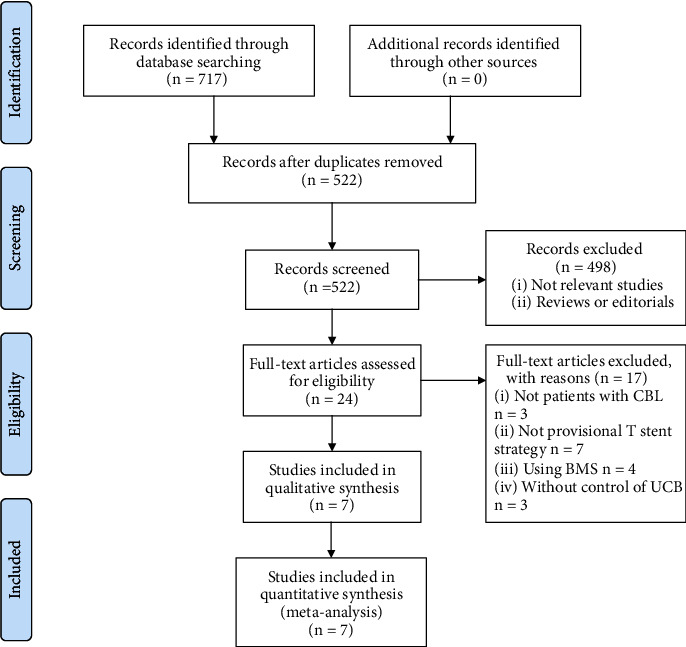
Flowchart of the database search.

The database search process is summarized in Figure 1. Briefly, 717 articles were found in the initial literature search of the databases; after excluding the duplications, 522 studies remained. An additional 498 were excluded through screening of the titles and abstracts mainly as they were irrelevant to the meta-analysis. The remaining 24 studies underwent a full-text review. Of these, 17 were further excluded for the reasons listed in Figure 1. Finally, seven studies [21–27] were included.

### 3.2. Characteristics of the Included Studies

The study characteristics are summarized in Table 1. Overall, four RCTs [22, 24, 25, 27] and three NRCTs [21, 23, 26] were included in the meta-analysis, comprising 803 patients with CBLs treated with provisional stenting using DES. These studies were published between 2013 and 2021 and were performed in Spain [21] and China [22–27]. The sample size of the included studies varied between 42 and 222. The mean ages of the included patients ranged from 56 to 64 years, and the proportion of men ranged from 57 to 83%. Paclitaxel-eluting balloons were used in six studies [21–24, 26, 27], and the type of DCB was not reported in the remaining study [25]. The mean follow-up durations varied between 6 and 12 months. For the four RCTs included, the details for random sequence generation were reported in two studies [22, 27] and the details for allocation concealment were reported in two studies [24, 27]. Blindness was not applied in any of the included RCTs, and the quality of the included RCTs was modest (Table 2). The quality of the included NRCTs were generally good, with NOS varying from 7 to 9 stars (Table 3).

### 3.3. Meta-Analysis Results

**Figure 2:**
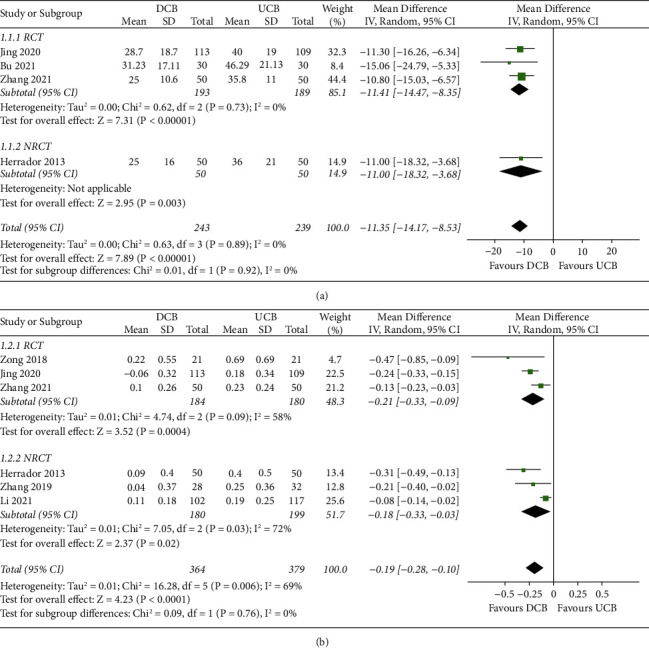
Forest plots for the effect of SB protection with DCB versus UCB on angiographic outcomes in patients with CBL treated with provisional stenting using DES. (a) Forest plots for the outcome of postoperative SB diameter stenosis. (b) Forest plots for the outcome of LLL of SB.

**Figure 3:**
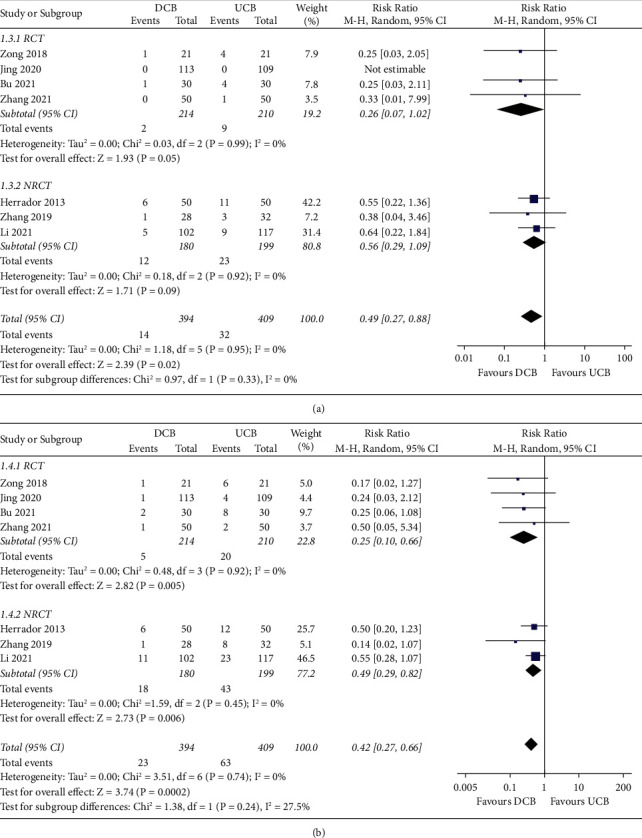
Forest plots for the effect of SB protection with DCB versus UCB on clinical outcomes in patients with CBL treated with provisional stenting using DES. (a) Forest plots for the outcome of TLR. (b) Forest plots for the outcome of MACE.

Pooled results showed that SB protection with DCB was associated with a lower extent of postoperative diameter stenosis (MD:−11.35%, 95% CI: −14.17 to−8.53, *p* < 0.001; I^2^ = 0%; Figure 2(a)) and less LLL (MD: −0.19 mm, 95% CI: −0.28 to−0.10, *p* < 0.001; I^2^ = 69%; Figure 2(b)) of SB as compared to that with UCB. Subgroup analysis according to the study design showed similar results (*p* for subgroup analyses = 0.92 and 0.76, respectively). Moreover, SB protection with DCB was associated with reduced risks of TLR (RR: 0.49, 95% CI: 0.27 to 0.88, *p*=0.02; I^2^ = 0%; Figure 3(a)) and MACEs (RR: 0.42, 95% CI: 0.27 to 0.66, *p* < 0.01; I^2^ = 0%; Figure 3(b)). Additionally, subgroup analysis showed consistent results in the meta-analyses of RCTs and NRCTs (*p*=0.33 and 0.24, respectively).

### 3.4. Publication Bias

**Figure 4:**
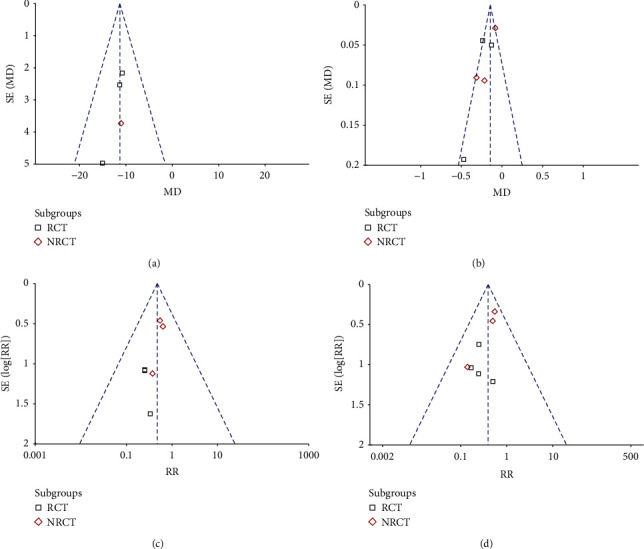
Funnel plots for the publication biases underlying the meta-analyses. (a) Postoperative diameter stenosis of SB. (b) LLL of SB. (c) TLR. (d) MACE.

Figures 4(a)–4(d) shows the funnel plots for the meta-analyses of the outcomes of SB diameter stenosis, LLL, incidence of TLR, and incidence of MACEs. Visual inspection confirmed the symmetry of the plots, which suggested low risks of publication biases. Egger's regression tests could not be performed due to the limited datasets available for each outcome.

## 4. Discussion

In this meta-analysis, we pooled the results of four RCTs and three NRCT. The results showed that compared to SB protection with UCB, the use of DCB was associated with less postoperative SB stenosis and smaller SB LLL for patients with CBLs treated with provisional stenting using DES. Moreover, we also found that the use of DCB was associated with significantly reduced incidences of TLR and overall MACEs in these patients as compared to UCB, with a follow-up duration of 6–12 months. Subgroup analyses according to the study design showed similar results in the meta-analyses of RCTs and NRCTs. Taken together, these results suggested that for patients with CBL treated with provisional stenting using DES, SB protection with DCB was associated with better angiographic and clinical outcomes than that with UCB. Although these results should be validated in large-scale RCTs with adequate sample sizes and longer follow-up durations, provisional stenting with DCB for SB protection may be applied as a promising treatment strategy for CBLs.

Some previous meta-analyses evaluated the role of DCB in the treatment of CBLs [33–35]. Although these studies generally suggested a favorable role of DCB in improving the angiographic and/or clinical outcomes in patients with CBLs, the inclusion criteria of the meta-analyses were relatively extensive, and significant clinical heterogeneity could be observed among the included studies [33–35]. These potential issues may make the interpretation of the results and application of the findings of the meta-analysis difficult in real-world clinical practice. For example, studies with different treatment strategies for the main vessels of the CBLs (with or without stent implantation in the main vessels) were included in two of the previous meta-analyses [34, 35]. Accordingly, the different treatment strategies for CBLs applied in the included studies may make the rationale for these meta-analyses less convincing. The meta-analyses also failed to show that DCB was associated with improved clinical outcomes in patients with CBLs despite the benefits observed in the angiographic results [34, 35]. Moreover, in another meta-analysis, studies using bare metal stents (BMS), DES, and mixed stent types were included, which may seriously confound the results regarding the effects of DCB on the clinical outcomes [33]. This is because the use of DES for the main vessels has been associated with reduced MACEs for CBLs, and this is attributed to repeated PCI in both the main vessels and SB [36, 37]. Compared to these studies, our study has a few strengths. Firstly, the inclusion criteria were relatively strict in our meta-analysis. We only considered studies of CBLs treated with provisional stenting using DES. This is because this treatment strategy has been applied in daily clinical practice, and studies evaluating the methods for POT of SB in this era are clinically important and applicable. Moreover, subgroup analyses according to the design characteristics of the studies were further performed, and consistent results were obtained for the meta-analyses of RCTs and NRCTs, which further demonstrated the robustness of the findings. Overall, the results of our meta-analysis expanded its therapeutic role as a strategy for SB protection of CBLs treated with provisional stenting using DES.

Our study also has limitations. Firstly, the included studies were relatively limited, had a moderate study quality, and included a small number of patients. Therefore, the results of the current meta-analysis should be validated in high-quality clinical trials in the future. In addition, most of the included studies were performed in China. Studies from other countries are also warranted. Moreover, the follow-up durations of the included studies varied between 6 and 12 months. The long-term benefits of DCB for SB protection of CBLs treated with provisional stenting using DES should be evaluated in the future. Finally, many factors besides the treatment strategy may affect the angiographic and clinical prognosis of patients with CBLs, such as the patient characteristics, lesion features, devices used, and experiences of the interventional cardiologists. The influences of these factors on the outcomes should also be investigated in the future.

In conclusion, the results of the meta-analysis indicated that for patients with CBL treated with provisional stenting using DES, SB protection with DCB was associated with better angiographic and clinical outcomes than those with UCB. These findings suggest that provisional stenting with DCB for SB protection may become an attractive treatment strategy for patients with CBLs.

## Figures and Tables

**Table 1:** Characteristics of the included studies.

Study	Country	Design	Number of patients	Mean age (years)	Male (%)	Stents in main vessels	DCB in SB	UCB in SB	Follow-up duration	Outcomes reported
Herrador 2013	Spain	NRCT	100	62.5	83	DES (taxus liberté)	Sequent® please paclitaxel eluting balloon	Conventional UCB	12 months	SB stenosis, LLL, TLR, and MACE

Zong 2018	China	RCT	42	56.3	57.1	DES	Sequent® please paclitaxel eluting balloon	Conventional UCB	6 months	LLL, TLR and MACE

Zhang 2019	China	NRCT	60	60.1	71.7	DES	Sequent® please paclitaxel eluting balloon	Conventional UCB	9 months	LLL, TLR, and MACE

Jing 2020	China	RCT	222	60.8	72.5	DES	Bingo® paclitaxel eluting balloon	Conventional UCB	9 months	SB stenosis, LLL, TLR, and MACE

Li 2021	China	NRCT	219	63.4	80.8	DES (promus premier)	Sequent® please paclitaxel eluting balloon	Conventional UCB	12 months	LLL, TLR, and MACE

Bu 2021	China	RCT	60	60.3	73.3	DES	DCB	Conventional UCB	12 months	SB stenosis, TLR, and MACE

Zhang 2021	China	RCT	100	58.5	61	Everolimus eluting stents	Sequent® please paclitaxel eluting balloon	Conventional UCB	9 months	SB stenosis, LLL, TLR, and MACE

RCT, randomized controlled trial; NRCT, nonrandomized controlled trial; DES, drug-eluting stent; DCB, drug-coated balloon; UCB, uncoated balloon; SB, side branch; LLL, late lumen loss; TLR, target vessel revascularization; MACE, major adverse cardiovascular events.

**Table 2:** Quality evaluation of the included RCTs.

Study	Random sequence generation	Allocation concealment	Blinding of participants	Blinding of outcome assessment	Incomplete outcome data addressed	Selective reporting	Other sources of bias	Total
Zong 2018	Low	Unknown	Unknown	Unknown	Low	Low	Low	4
Jing 2020	Unknown	Low	Unknown	Unknown	Low	Low	Low	4
Bu 2021	Unknown	Unknown	Unknown	Unknown	Low	Low	Low	3
Zhang 2021	Low	Low	Unknown	Unknown	Low	Low	Low	5

**Table 3:** Quality evaluation of the included NRCT.

Study	Representativeness of the exposed cohort	Selection of the nonexposed cohort	Ascertainment of exposure	Demonstration that outcome of interest was not present at start of study	Comparability - age and gender	Comparability - other factors	Assessment of outcome	Was follow-up long enough for outcomes to occur	Adequacy of follow-up of cohorts	Total
Herrador 2013	0	1	1	1	1	1	1	1	1	8
Zhang 2019	0	1	1	1	1	1	1	0	1	7
Li 2021	1	1	1	1	1	1	1	1	1	9

## Data Availability

The datasets generated and analyzed during the current study are included in the article, further inquiries can be directed to the corresponding author.
